# Targeting Sphingosine Kinase by ABC294640 against Diffuse Intrinsic Pontine Glioma (DIPG)

**DOI:** 10.7150/jca.46269

**Published:** 2020-05-22

**Authors:** Lu Dai, Jungang Chen, Zhen Lin, Zhaoxiong Wang, Shengyu Mu, Zhiqiang Qin

**Affiliations:** 1 Departments of Pathology, Winthrop P. Rockefeller Cancer Institute, University of Arkansas for Medical Sciences, 4301 W Markham St, Little Rock, AR 72205, USA.; 2 Department of Pathology, Tulane University Health Sciences Center, Tulane Cancer Center, 1700 Tulane Ave., New Orleans, LA 70112, USA.; 3 Pharmacology & Toxicology, Winthrop P. Rockefeller Cancer Institute, University of Arkansas for Medical Sciences, 4301 W Markham St, Little Rock, AR 72205, USA.

**Keywords:** sphingolipid, ceramide, brain tumor, DIPG, pediatric cancer

## Abstract

As a highly aggressive pediatric brainstem tumor, diffuse intrinsic pontine glioma (DIPG) accounts for 10% to 20% of childhood brain tumors. The survival rate for DIPG remains very low, with a median survival time as less than one year even under radiotherapy, the current standard treatment. Moreover, over than 250 clinical trials have failed when trying to improve the survival compared to radiotherapy. The sphingolipid metabolism and related signaling pathways have been found closely related to cancer cell survival**;** however, the sphingolipid metabolism targeted therapies have never been investigated in DIPG. In the current study, the anti-DIPG activity of ABC294640, the only first-in-class orally available Sphingosine kinase (SphK) inhibitor was explored. Treatment with ABC294640 significantly repressed DIPG cell growth by inducing intracellular pro-apoptotic ceramides production and cell apoptosis. We also profiled ABC294640-induced changes in gene expression within DIPG cells and identified many new genes tightly controlled by sphingolipid metabolism, such as IFITM1 and KAL1. These genes are required for DIPG cell survival and display clinical relevance in DIPG patients' samples. Together, our findings in this study indicate that targeting sphingolipid metabolism may represent a promising strategy to improve DIPG treatment.

## Introduction

Diffuse intrinsic pontine glioma (DIPG) is a highly aggressive pediatric brainstem tumor that accounts for about 10% to 20% of childhood brain tumors 1-3. These tumors are mostly seen in children between 5 and 10 years old, but have been reported to occur at any age of childhood. The survival rate for DIPG remains very low, with a median survival time as less than one year even under radiotherapy, the current standard treatment. Moreover, more than 250 clinical trials have failed when trying to improve the survival compared to radiotherapy 4-6. For example, some studies have reported chemotherapy has failed to show benefits beyond radiotherapy for improving DIPG patients' survival 5. Moreover, the sensitive location of these tumors and lack in surgical specimens (or even lack in the patient-derived cell lines) have hindered our understanding of the DIPG development, pathogenesis as well as discovery of effective targeted therapy. We and others recently have reported some new therapeutic targets and agents against DIPG, including natural products (e.g., Brefeldin A, Combretastatin A4); Emtansine conjugated to peptide nanofiber precursor (DM1-NFP); synthesized novel platinum (ii) complexes; combined AXL and HDAC inhibition 7-12. However, most of these new treatments are still at preclinical stage, which require clinical trials to assess their efficacy and tolerability. Unfortunately, a very recent Phase 2 study reported that addition of valproic acid (VPA) and bevacizumab to radiation failed to improve the survival of DIPG patients 13.

The ceramidase in sphingolipid metabolism is responsible for hydrolytic conversion of ceramide to sphingosine, which is then phosphorylated by SphK1 or SphK2, two isoforms of sphingosine kinases to generate sphingosine-1-phosphate (S1P) 14. In fact, the cellular balance of ceramide and S1P can determine the fate of tumor cells, with the accumulation of ceramides favoring cell apoptosis, while the accumulation of S1P favoring cell proliferation 14,15. Among sphingolipid metabolic, ceramide has been considered as a central lipid mediator with tumor suppression function, because it can tightly regulate many cellular signaling related to apoptosis, cell cycle and autophagy 14,16. Due to their pleiotropic roles, targeting bioactive sphingolipids has recently evolved as a promising therapeutic approach for improving cancer treatment 17. As a key enzyme of sphingolipid metabolism, a highly selective small molecule inhibitor of SphK, 3-(4-chlorophenyl)-adamantane-1-carboxylic acid (pyridin-4-ylmethyl)amide (named as ABC294640), has been recently developed 18,19, and shows significant anti-tumor effects on a variety of cancers 20-24. Moreover, Britten *et al.* have reported the promising results from a Phase I clinical trial about ABC294640 in patients with advanced solid tumors 25. They reported that ABC294640 at 500 mg bid was well tolerated by these cancer patients and achieved biologically relevant concentrations in their plasma 25.

Our group reported that targeting sphingolipid metabolism with either ABC294640 or exogenous ceramides resulted in *in vitro* and *in vivo* anticancer activities for virus-associated malignancies 20,26-28, as well as non-small cell lung cancer (NSCLC) 29. However, the functional role of sphingolipid metabolism and related cellular network in DIPG remains almost unknown. Even ABC294640 has displayed broad anti-tumor activities in a variety of cancers, we think that the underlying mechanisms especially sphingolipid related cellular contents are tumor type-dependent. It is also unclear whether the sphingolipid metabolism targeted therapies can be developed for improving DIPG treatment. In the current study, we investigated the response of DIPG cells to SphK inhibition by ABC294640, identified new cellular genes controlled by sphingolipid metabolism in DIPG cells and validated their functions in DIPG pathogenesis. Our results provide new insights into the mechanism and potential utility of targeting sphingolipid metabolism in a deadly form of pediatric cancer.

## Materials and Methods

### Cell culture and reagents

The DIPG cell lines SF8628 and SF7761 that harbor the histone H3.3 Lys 27-to-methionine (K27M) mutation were purchased from Millipore-Sigma and cultured as recommended by the manufacturer. The cortical neuronal cell-line, HCN-2, was purchased from American Type Culture Collection (ATCC), and cultured as recommended by the manufacturer. All the experiments were carried out using cells harvested at low (<20) passages. ABC294640 was purchased from SelleckChem.

### Cell proliferation and apoptosis assays

Cell proliferation was determined by using the WST-1 assays (Roche) according to the manufacturer's instructions. Briefly, after the period of treatment of cells, 10 μL/well of cell proliferation reagent, WST-1 (4-[3-(4-Iodophenyl)-2-(4-nitro-phenyl)-2H-5-tetrazolio]-1,3-benzene disulfonate), was added into 96-well microplate and incubated for 3 h at 37 °C in 5% CO_2_. The absorbance of samples was measured by using a microplate reader at 490 nm. Flow cytometry was used to the quantitative analysis of apoptosis with the FITC-Annexin V/propidium iodide (PI) Apoptosis Detection Kit I (BD Pharmingen).

### Soft agar assays

The anchorage-independent growth ability was determined using the soft agar assays as described previously 30. Briefly, a base layer containing 0.5% agarose medium and 5% FCS was poured into the six-well plates. Then, 2,000 cells were mixed with 0.4% agarose in Dulbecco's Modified Eagle Medium (DMEM) containing 5% (v/v) FCS to form a single-cell suspension. The plates were then incubated for 4-5 weeks at 37 °C. Colonies were stained with 0.005% (w/v) crystal violet and photographed under a phase-contrast microscope.

### Immunoblotting

Total cell lysates (20 μg) were resolved by 10% (w/v) SDS-PAGE, transferred to nitrocellulose membranes, and immunoblotted with antibodies for cleaved Caspase3, cleaved PARP, Bax, XIAP, SphK1, SphK2 (Cell Signaling), IFITM1, KAL1 (Abcam) and β-Actin or Tubulin (Sigma) for the loading controls. Immunoreactive bands were identified using an enhanced chemiluminescence reaction (Perkin-Elmer), and visualized by the autoradiography.

### Sphingolipid analyses

Quantification of sphingolipid species was performed using a Thermo Finnigan TSQ 7000 triple-stage quadruple mass spectrometer (Thermo Fisher Scientific). Quantification was based on calibration curves generated by spiking an artificial matrix with known amounts of target standards and an equal amount of the internal standard. The ratio of sphingolipid normalized to total phospholipid phosphate level was shown as final results using the Bligh and Dyer lipid extract method as described previously 31.

### RNA-Sequencing and analysis

RNA-Sequencing for each sample was performed in biological triplicate using the Genome Analyzer IIx (Illumina) at LSUHSC Translational Genomics Core (TGC) Facility. The completed RNA-Sequencing data has been submitted to NCBI Sequence Read Archive and available (SRA, accession # PRJNA565990). Raw sequencing reads were analyzed using the RSEM software (version 1.3.0; human GRCh38 genome sequence and annotation) for quantification of human gene expression as previously described.25 The EBSeq software was utilized to analyze statistically differentially expressed genes using a false discovery rate (FDR) < 0.05. Differentially expressed genes between the ABC294640- and vehicle-treated cells were used as input for the Ingenuity Pathway Analysis (IPA)'s downstream effects analyses including the canonical pathway analysis and disease function analysis. The Z-score was calculated by the Z-score algorithm of the IPA, which can predict the direction of a biological function change. RNA-Sequencing data of DIPG tumor samples and paired normal brain tissues were obtained from the National Institutes of Health (NIH) Genotypes and Phenotypes (dbGaP) database under the accession number (SRA# SRP136329) after obtaining the NIH permission. Transcript quantification of human genes was conducted as previously described 32.

### RNA interference (RNAi) assays

For RNAi assays, *IFITM1*, *KAL1, SphK1 or SphK2* On-Target plus Smart pool siRNA (Dharmacon) or negative control siRNA were delivered at the concentration of 10 or 25 nM, using the DharmaFECT transfection reagent as recommended by the manufacturer.

### Statistical analysis

The two-tailed Student's t-test was used to determine the significance for differences between the experimental and control groups.

## Results

### ABC294640 displays effective anti-DIPG activity

**Figure 1:**
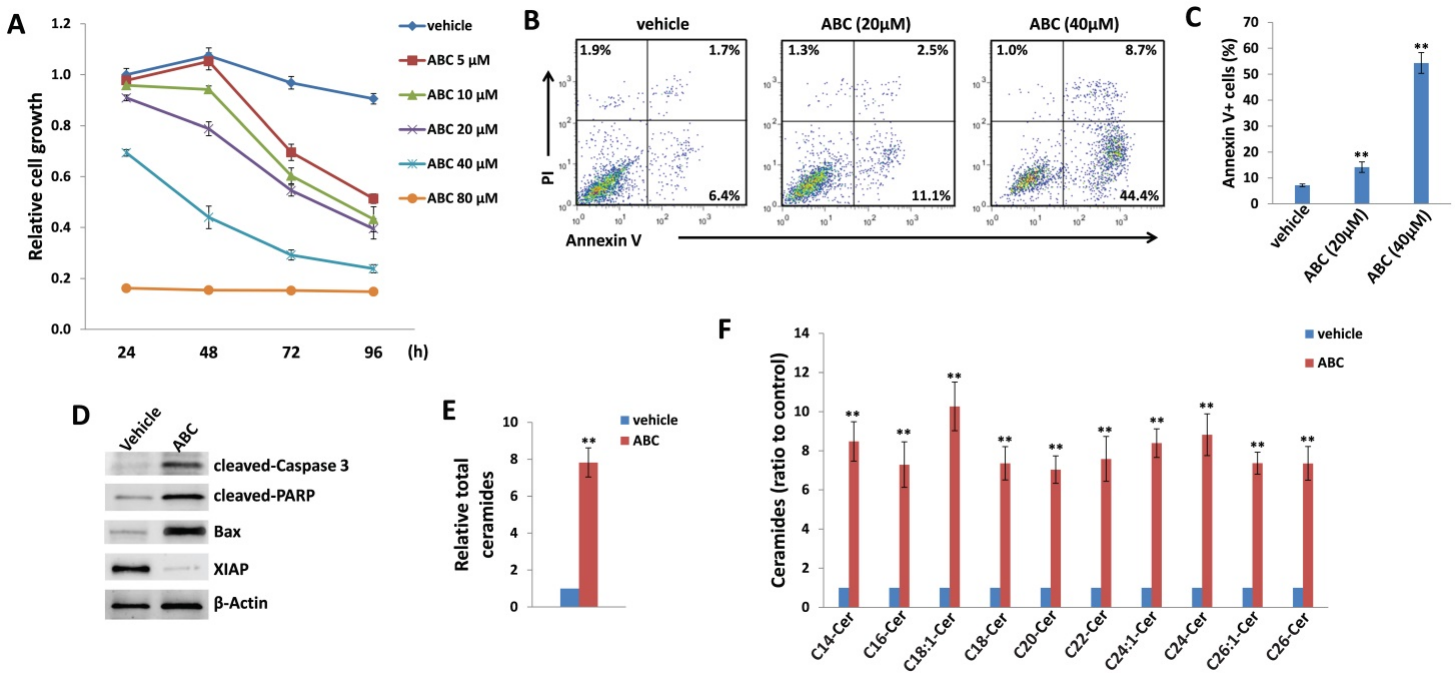
** ABC294640 treatment effectively reduces DIPG proliferation through inducing tumor cell apoptosis and intracellular ceramides production.** (**A**) DIPG cell line SF8628 were treated with the indicated concentrations of ABC294640 or vehicle for 24-96 h. The cell proliferation status was then examined using the WST-1 cell proliferation assays (Roche). (**B-C**) SF8628 were treated with the indicated concentrations of ABC294640 or vehicle for 24 h. Cell viability and apoptosis were then measured by Annexin V-PI staining and flow cytometry analysis. Error bars represent S.D. for 3 independent experiments. (**D**) Protein expression was detected using immunoblots. (**E-F**) SF8628 were treated with 40 µM of ABC294640 or vehicle for 24 h. Ceramide species and total levels were then quantified using lipidomics analyses as described in the Methods. Error bars represent S.D. for 2 independent experiments. ** = p< 0.01.

As mentioned above, currently there are limited patient-derived DIPG cell lines commercially available. Here we used two DIPG cell lines, SF8628 and SF7761, both of which are derived by surgical biopsies from H3.3K27M DIPG patients 33. H3.3K27M, a somatic mutation of histone H3.3 resulting in a lysine 27 to methionine substitution occurs in 60% of DIPG 34. In H3.3K27M DIPG patient samples, the levels of H3K27 dimethylation (H3K27me2) and trimethylation (H3K27me3) are reduced globally. These epigenetic changes are thought to be important factors driving DIPG oncogenesis 34, 35. In a time-course “killing” assay, we found that ABC294640 treatment effectively reduced SF8628 cell growth in a dose-dependent manner when compared to the vehicle-treated controls (**Figure 1A**). We also observed similarly inhibitory effects of ABC294640 on SF7761 cell line (**Figure S1**). In contrast, ABC294640 showed much less inhibitory effects on the growth of normal brain/neuronal cell-line, HCN-2, with only 80 μM concentration showing limited inhibition (**Figure S2**). Even though both DIPG cell lines carrying H3.3K27M, they display distinct morphology in cultures, especially SF7761 having much less adhesive capability, easily clustering (not good for WST-1 assays measurement), and difficultly being cultured (**Figure S3**), so we decided to only use SF8628 for subsequent experiments. We next sought to determine the mechanisms for anti-DIPG activity of ABC294640. Using flow cytometry, we found that ABC294640 treatment induced apoptosis of DIPG cells in a dose-dependent fashion, including the increased subpopulation of both early (Annexin V+/PI-) and late (Annexin V+/PI+) apoptotic cells (**Figure 1B-C**). We also observed the increased cleavage of caspases-3 and PARP, and the expression of pro-apoptotic protein, Bax, from ABC294640-treated DIPG cells (**Figure 1D**). In contrast, we observed a decreased expression of X-linked inhibitor of apoptosis protein (XIAP), which is a physiologic substrate of Akt, and its function is stabilized to inhibit programmed cell death and having a direct effect on caspases 36. These results are consistent with the notion that ABC294640 exerts an anti-proliferative effect on DIPG cells by inducing programmed cell death.

### ABC294640 induces intracellular ceramides production from DIPG cells by lipidomics analysis

Next, we sought to determine if these effects were related to changes in sphingolipid metabolism. Mass spectrometric (MS)-based lipidomics analysis were used to quantify and compare intracellular levels of bioactive ceramide species in the SF8628 cell line treated with or without ABC294640. We found that ABC294640 increased total levels of intracellular ceramides (~8 folds) in DIPG cells when compared to the vehicle group. The lipidomics analysis showed that most of individual ceramide species including those from C14-Cer to C26-Cer were upregulated in ABC294640-treated DIPG cells, although the extent of the increase varies among the ceramide species (**Figure 1E-F**). The composition and proportion of ceramide species within SF8628 cells with or without ABC294640 treatment were calculated (**Figure S4**). The predominant ceramide signature within DIPG cells included C16-, C22- and C24:1-Cer species. Interestingly, we found that ABC294640 treatment did not significantly alter the proportion of ceramide species in DIPG cells, which is different from what we have observed in virus-associated tumors and lung cancers 26,28,29.

### Transcriptomic analysis of gene profiling altered within ABC294640-treated DIPG cells

**Figure 2:**
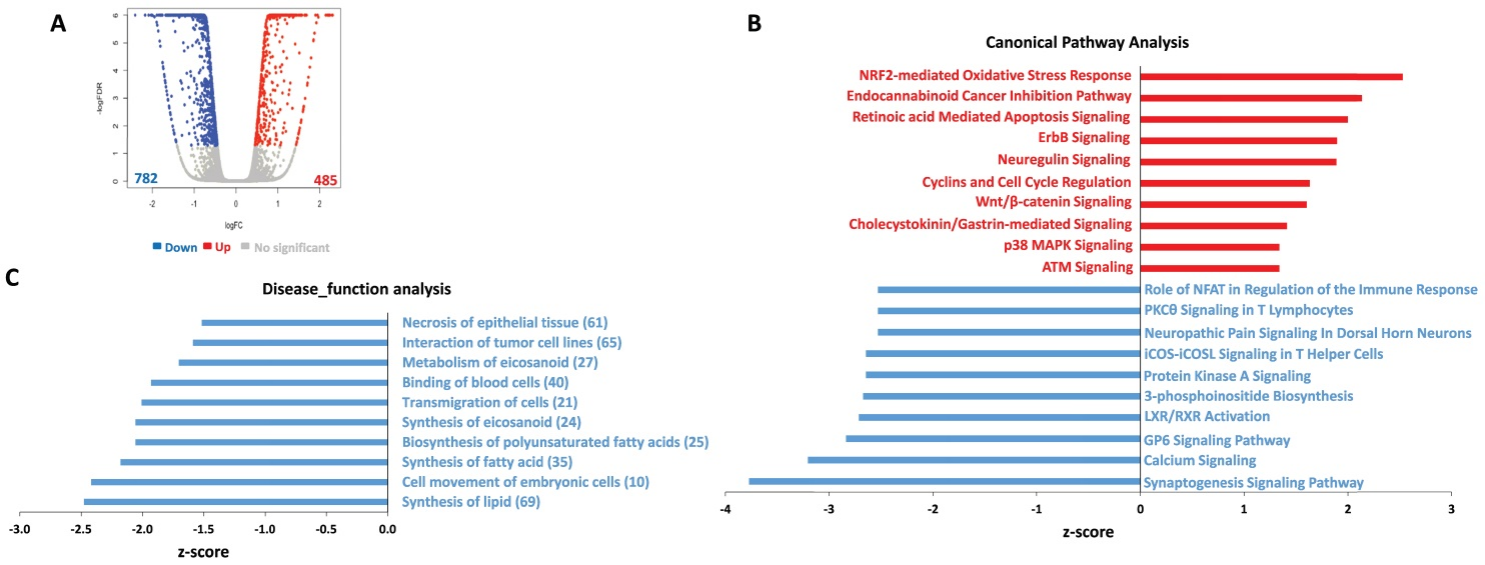
** Ingenuity pathway analysis (IPA) of global gene profile altered by ABC294640 from DIPG cells.** (**A**) The RNA-Sequencing (Illumina) was used to investigate the transcriptome change between ABC294640 and vehicle treated DIPG SF8628 cells. The significantly changed genes (expression change ≥ 2-fold and p<0.05) were shown in the Volcano plot panels. (**B-C**) The top 10 activated (red) and 10 inhibited (blue) canonical pathways (or Disease_functions) discovered in the ABC294640-treated cells by the IPA. The pathways (B)/functions (C) were ranked by the Z-score. The Z-score is a value calculated by the Z-score algorithm of the IPA. The Z-score is utilized to predict the direction of change for a biological function: if it is increased, the Z-score is > 0; if it is decreased, the Z-score is < 0.

To determine the global metabolic and cellular changes induced by ABC294640, we compared the global gene profile altered between vehicle- and ABC294640**-**treated tumor cells by using RNA-Sequencing (Illumina) analysis. We first found 485 genes significantly upregulated and 782 genes significantly downregulated (≥ 2 folds and FDR< 0.05) in ABC294640**-**treated SF8628 cell line, respectively (**Figure 2A**). The top 20 significantly upregulated and downregulated candidates were listed in **Table 1**. Interestingly, most of these candidates, their functions have never been reported in DIPG cells. Ingenuity pathway analysis (IPA) analysis of altered gene profiling indicated that many canonical pathways and disease_function categories within DIPG cells were affected by ABC294640 treatment (**Figure 2B-C**). For example, several canonical pathways related to cancer cell survival, such as retinoic acid mediated apoptosis signaling, cyclins and cell cycle regulation, and calcium signaling were impacted by treatment with ABC294640. Since ABC294640 is a selective SphK inhibitor, it is not surprising to see several fatty acid/lipid synthesis categories negatively regulated by ABC294640.

### Sphingolipid metabolism regulation of IFITM1 and KAL1 which are required for DIPG cell survival

To functionally validate those significantly altered genes within ABC294640-treated DIPG cells discovered by RNA-Sequencing, we selected two candidates, IFITM1 and KAL1, from the list. Interferon-induced transmembrane protein 1 (IFITM1) is originally identified as part of membrane complexes transducing homotypic adhesion signals in lymphocytes 37,38. The expression of IFITM1 is induced by IFN-α and/or IFN-γ (to a lesser extent), and has been reported in several types of cancer cells 39,40. Anosmin-1, encoded by the KAL1 gene, is an important extracellular matrix (ECM) component that plays essential roles in the establishment of olfactory and gonadotrophin-releasing hormone (GNRH) neurons during early brain development. Loss-of-function mutations of KAL1 results in Kallmann syndrome with delayed puberty and anosmia 41. However, their functions in DIPG cells remain unclear.

**Figure 3:**
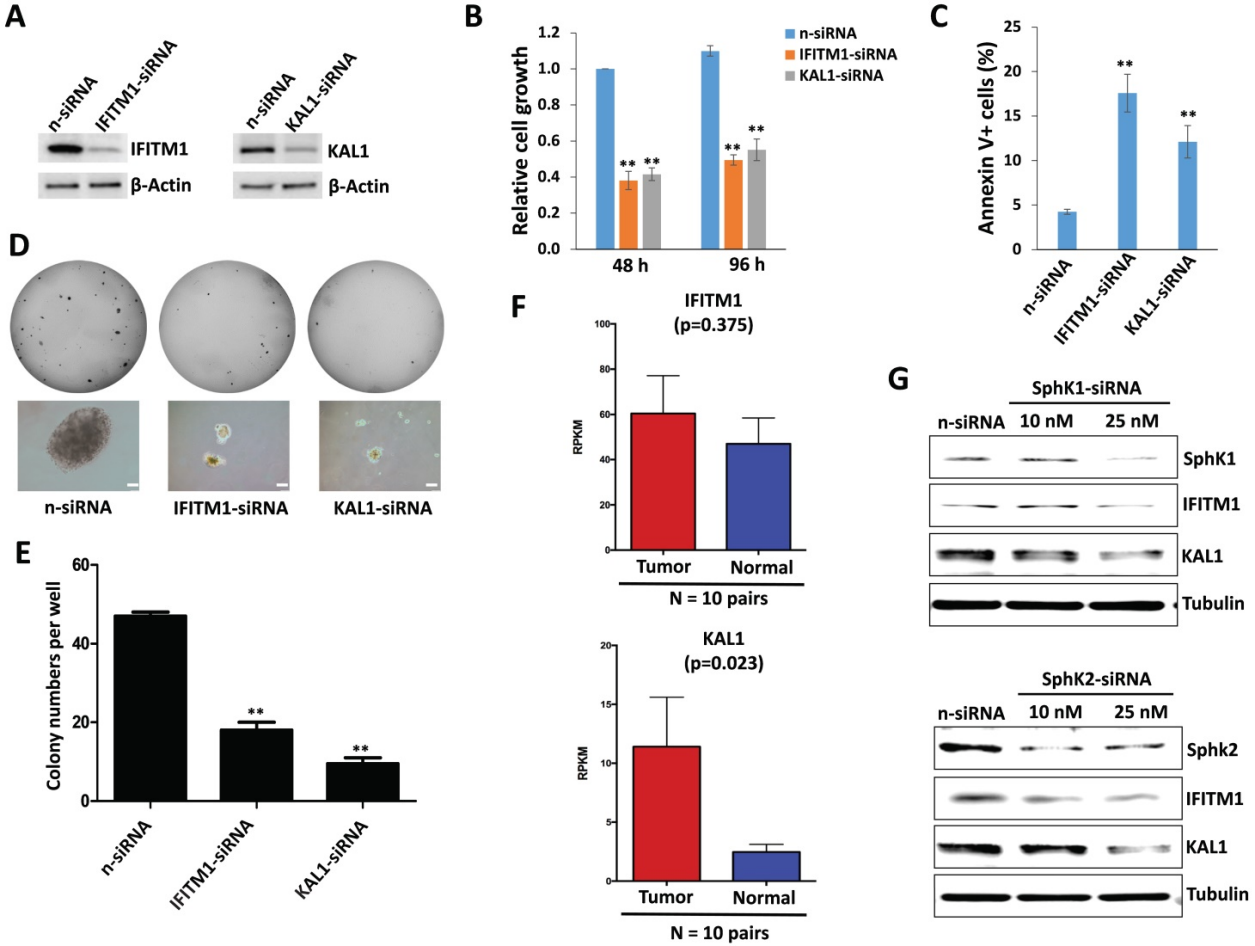
** Sphingolipid metabolism regulation of IFITM1 and KAL1 expression is essential for DIPG cell survival and growth.** (**A-C**) SF8628 were transfected with *IFITM1*-siRNA, *KAL1*-siRNA or non-target control siRNA (n-siRNA) for 48-96 h, then the protein expression, cell proliferation and apoptosis were measured as described above. (**D-E**) The anchorage-independent growth ability was determined using the soft agar assays. Error bars represent S.D. for 3 independent experiments. ** = p< 0.01. (**F**) The expression of IFITM1 and KAL1 in 10 DIPG and paired normal brain tissues. RPKM (Reads Per Kilobase of transcript per Million mapped reads) values were calculated using reads across the gene exons. Data were represented as the mean (+/-) SEM. (**G**) SF8628 were transfected with *SphK1*-siRNA, *SphK2*-siRNA or non-target control siRNA (n-siRNA) for 72 h, then the protein expression was measured by immunoblots.

We observed the downregulation of both IFITM1 and KAL1 in DIPG cells treated with ABC294640 (**Table 1**). We next found that silencing of either IFITM1 or KAL1 by RNAi effectively repressed DIPG cell growth, inducing tumor cell apoptosis in a manner similar to treatment with ABC294640 (**Figure 3A-C**). RNAi against either IFITM1 or KAL1 also inhibited anchorage-independent growth of DIPG cells (**Figure 3D-E**), again consistent with the results obtained in experiments with the SphK inhibitor.

Currently, a DIPG clinical database is not publically available. Recently, we have obtained the permission to access RNA-Sequencing data of DIPG tumor samples and paired normal brain tissues from the NIH Genotypes and Phenotypes (dbGaP) database. Here we found that both IFITM1 and KAL1 expression were higher in DIPG tumor samples than in their paired normal brain tissues, although only KAL1 proved to be statistically significant probably due to the small number of collection (**Figure 3F**). Together, these data support a role for IFITM1 and KAL1 in promoting DIPG survival or growth.

To further understand the connection of IFITM1 and KAL1 with sphingolipid metabolism in DIPG cells, we silenced SphK1 and SphK2 in SF8628, two of major enzymes in sphingolipid metabolism using RNAi, respectively. We found that knockdown of either SphK1 or SphK2 effectively reduced IFITM1 and KAL1 expression (**Figure 3G**) indicating sphingolipid metabolism indeed regulates these cellular genes expression and functions to determine tumor cell survival.

## Discussion

As a rare but highly aggressive pediatric cancer, DIPG is currently lacks an effective treatment, making prognosis very poor. Our efforts seek to identify new therapeutic targets and develop promising strategies for maximally prolonging the survival of patients. In the current study, we used one of few commercially available DIPG patient-derived cell lines (SF8628) to determine if targeting sphingolipid metabolism is a useful strategy for improving patient outcomes. We found that treatment with ABC294640, the only first-in-class orally available inhibitor of SphK, selectively repressed DIPG cell growth through inducing intracellular pro-apoptotic ceramides production and tumor cell apoptosis. In general, there are three major ceramide generation pathways: the sphingomyelinase pathway (sphingomyelin→ceramide); the *de novo* pathway (3-keto-dihydrosphingosine→dihydro sphingosine→dihydroceramide→ceramide); and the Salvage pathway (S1P→sphingosine →ceramide) 16,42. MS-based lipidomics analyses will be used to further determine which pathways mediate intracellular ceramides production from ABC294640-treated DIPG cells. Ceramide synthases (CerSs) are the enzymes responsible for ceramide generation in the *de novo* and Salvage pathways. Currently, six different CerSs have been identified in mammary cells, CerS1-6 43, and different isoforms of CerS generate an array of ceramide species with distinct length of fatty acid chain 44. Our previous study showed that ABC294640 treatment increased the gene transcription and protein expression for CerS2, CerS4 and CerS6 from some virus-associated lymphomas 26. However, the signature of ceramide species and regulation of ceramide production are potentially differentiated in different types of cancer cells. For example, we found that ABC294640 treatment did not alter the proportion of ceramide species in DIPG cells, in contrast to the significant changes we observed previously in virus-associated tumors and lung cancers 26,28,29. In addition, we previously reported that ABC294640 treatment induced dihydro(dh)-ceramide production in virus-associated tumors and lung cancers, which also contributed to “killing” of tumor cells 20,28,29. In contrast, we found low or undetectable levels of dh-ceramide species in DIPG cells even after exposure to ABC294640 (data not shown), implying that dh-ceramides are not involved in ABC294640-mediated DIPG cell death.

Although the functional role of sphingolipid metabolism (especially ceramide production) and targeted therapy have not been reported in DIPG, there are some related reports in other brain tumors. One recent study reported that the overexpression of CerS1 increased C18-Cer level and led to lethal autophagy in human glioma cells 45. Another recent study revealed that co-delivery of tumor-derived exosomes with α-galactosylceramide (α-GalCer) on a dendritic cell (DC)-based vaccine showed excited effects on glioblastoma immunotherapy, inducing strong activation of tumor-specific cytotoxic T lymphocytes, synergistically breaking immune tolerance and improving the immunosuppressive environment *in vivo* 46. In one review article, Sordillo *et al* postulated that some chemotherapeutic agents or radiotherapy may induce short-term responses in glioblastoma patients by increasing ceramide levels, however, the SphK may cause the increased ceramide to be metabolized to S1P, therefore restoring the abnormally high S1P to ceramide balance and representing part of the reason for the nearly 100% recurrence rate of glioblastoma 47. Thus, the use of maintenance therapy with a SphK inhibitor in patients with glioblastoma who have tumor reduction or stable disease after therapy should be investigated and considered.

In addition to the regulation of intracellular ceramides production, sphingolipid metabolism actually regulates many other cellular factors related to cell survival or death. Our recent transcriptomic analyses revealed that a subset of tumor suppressor genes (~25 genes) were significantly upregulated in virus-associated lymphomas by exogenous dhC16-Cer 27, which may control tumor cell growth. In this study, we have identified new genes within DIPG affected by ABC294640. Two of these genes, IFITM1 and KAL1, were selected and found to have a strong impact on DIPG cell survival with clinical relevance. Interestingly, Balbous *et al* reported IFITM1 as one of mesenchymal glioma stem cell makers responsible for cell invasion and gliomasphere initiation, which also revealed strong correlation with overall survival of glioblastoma patients 48. Several studies reported overexpression of IFITM1 in some types of tumors, such as colorectal, gastrointestinal and breast cancers 49,50. Furthermore, these studies demonstrated a positive correlation between IFITM1 overexpression and tumor progression, too. For another gene, KAL1, Choy *et al* found that its mRNA level was significantly upregulated in high-grade primary brain tumors when compared to the normal brain and/or low-grade tumors 51. They also found that KAL1 enhanced glioblastoma cells proliferation and motility *in vitro*, through forming a complex with integrin β1 induced downstream signaling, and modulating cell adhesion 51. Interestingly, we found that both SphK1 and SphK2 could regulate IFITM1 and KAL1 expression, although ABC294640 has much less impact on SphK1 when compared to SphK2. Thus our data demonstrate that sphingolipid metabolism indeed regulates the expression of other cellular factors related to DIPG survival such as IFITM1 and KAL1, while the regulatory mechanisms still require further investigation.

In conclusion, our data for the first time demonstrate sphingolipid metabolism controls DIPG cell survival, which may represent a promising target against this rare but deadly pediatric brain tumor. Our findings also provide new evidence that sphingolipid metabolism regulates additional cellular factors to affect DIPG cell survival and growth. Although its promise in DIPG treatment, there are still many challenging for developing sphingolipid metabolism targeted therapy including ABC294640. One of remained questions is the permeability and uptake of ABC294640 to blood-brain barrier (BBB) and blood-tumor barrier (BTB), which has never been tested before. Also, the *in vivo* efficacy of ABC294640 or other sphingolipid-related agents need to be assessed by using DIPG intracranial animal models in future studies.

## Supplementary Material

Supplementary figures and tables.

## Figures and Tables

**Table 1:** The top 20 candidate genes upregulated or downregulated in DIPG cells treated by ABC294640

Gene Symbol	Description	Ratio
TM4SF19-TCTEX1D2	TM4SF19-TCTEX1D2	203.2956944
SERPINB2	Serpin family B member 2	198.8279343
CXCL8	C-X-C motif chemokine ligand 8	175.8729164
AP003419.11	AP003419.11/novel protein	166.9185386
KRT34	Keratin 34	163.7514827
RP11-466H18.1	Ribosomal protein L36a pseudogene	153.5685184
ANKRD22	Ankyrin repeat domain 22	139.5586948
CH507-9B2.4	CH507-9B2.4/novel protein	92.99857464
SPINK1	Serine peptidase inhibitor, Kazal type 1	88.91478078
SLC7A11-AS1	SLC7A11 antisense RNA 1	83.299466
ANGPTL4	Angiopoietin like 4	79.47843571
CORO7-PAM16	CORO7-PAM16	75.18388531
CTD-2066L21.3	CTD-2066L21.3/lincRNA	71.37901957
AC068533.7	AC068533.7/novel protein	70.82918348
RP11-1151B14.4	Novel transcript, antisense to ALPK2	62.40569457
CXorf67	Chromosome X open reading frame 67	58.1829534
IL36B	Interleukin 36, beta	53.78426467
TNFSF12-TNFSF13	TNFSF12-TNFSF13	51.2770121
GPR78	G protein-coupled receptor 78	51.25501866
RP11-613M10.8	RP11-613M10.8/novel protein	50.4192678
COL3A1	Collagen type III alpha 1 chain	0.003891426
IFITM1	Interferon induced transmembrane protein 1	0.006783706
LGALS2	Galectin 2	0.009437712
HEYL	Hes related family bHLH transcription factor with YRPW motif-like	0.0097645
RP11-588K22.2	Novel transcript, overlapping GUCY1A3	0.01022648
AC093850.2	AC093850.2/novel transcript	0.011349859
DUSP27	Dual specificity phosphatase 27	0.012750501
TLL1	Tolloid like 1	0.012939696
RP11-680F20.6	V-set and immunoglobulin domain-containing protein 10-like	0.013194198
C1QTNF3-AMACR	C1QTNF3-AMACR	0.013937263
APOC1	Apolipoprotein C1	0.014431779
CDH23	Cadherin related 23	0.015076107
TEX41	Testis expressed 41	0.015646885
KAL1	Anosmin 1	0.015662941
COL5A3	Collagen type V alpha 3 chain	0.016356182
DLX3	Distal-less homeobox 3	0.017533291
RP11-598P20.5	RP11-598P20.5/novel protein	0.017621652
ZMYND15	Zinc finger MYND-type Containing 15	0.017651757
MUC7	Mucin 7, secreted	0.017651757

## References

[1] Long W, Yi Y, Chen S, Cao Q, Zhao W, Liu Q (2017). Potential New Therapies for Pediatric Diffuse Intrinsic Pontine Glioma. Front Pharmacol.

[2] Mohammad F, Weissmann S, Leblanc B, Pandey DP, Hojfeldt JW, Comet I (2017). EZH2 is a potential therapeutic target for H3K27M-mutant pediatric gliomas. Nat Med.

[3] Lapin DH, Tsoli M, Ziegler DS (2017). Genomic Insights into Diffuse Intrinsic Pontine Glioma. Frontiers in Oncology.

[4] Piunti A, Hashizume R, Morgan MA, Bartom ET, Horbinski CM, Marshall SA (2017). Therapeutic targeting of polycomb and BET bromodomain proteins in diffuse intrinsic pontine gliomas. Nat Med.

[5] Gwak HS, Park HJ (2017). Developing chemotherapy for diffuse pontine intrinsic gliomas (DIPG). Crit Rev Oncol Hemat.

[6] Pollack IF, Agnihotri S, Broniscer A (2019). Childhood brain tumors: current management, biological insights, and future directions. J Neurosurg-Pediatr.

[7] Chen J, Lin Z, Barrett L, Dai L, Qin Z (2020). Identification of new therapeutic targets and natural compounds against diffuse intrinsic pontine glioma (DIPG). Bioorganic Chemistry.

[8] Bellat V, Alcaina Y, Tung C-H, Ting R, Michel A, Souweidane M (2020). A Combined Approach of Convection-Enhanced Delivery of Peptide Nanofiber Reservoir to Prolong Delivery of DM1 for Diffuse Intrinsic Pontine Glioma Treatment. Neuro-Oncology.

[9] Boulet MHC, Marsh LK, Howarth A, Woolman A, Farrer NJ (2020). Oxaliplatin and [Pt(R,R-DACH)(panobinostat-2H)] show nanomolar cytotoxicity towards diffuse intrinsic pontine glioma (DIPG). Dalton Transactions.

[10] Meel MH, de Gooijer MC, Metselaar DS, Sewing ACP, Zwaan K, Waranecki P (2019). Combined therapy of AXL and HDAC inhibition reverses mesenchymal transition in diffuse intrinsic pontine glioma. Clin Cancer Res. 2020: clincanres.3538.

[11] Kluiver TA, Alieva M, van Vuurden DG, Wehrens EJ, Rios AC (2020). Invaders Exposed: Understanding and Targeting Tumor Cell Invasion in Diffuse Intrinsic Pontine Glioma. Frontiers in Oncology.

[12] Bailey CP, Figueroa M, Mohiuddin S, Zaky W, Chandra J (2018). Cutting Edge Therapeutic Insights Derived from Molecular Biology of Pediatric High-Grade Glioma and Diffuse Intrinsic Pontine Glioma (DIPG). Bioengineering.

[13] Su JM, Murray JC, McNall-Knapp RY, Bowers DC, Shah S, Adesina AM (2020). A phase 2 study of valproic acid and radiation, followed by maintenance valproic acid and bevacizumab in children with newly diagnosed diffuse intrinsic pontine glioma or high-grade glioma. Pediatr Blood Cancer.

[14] Ogretmen B, Hannun YA (2004). Biologically active sphingolipids in cancer pathogenesis and treatment. Nat Rev Cancer.

[15] Cuvillier O, Pirianov G, Kleuser B, Vanek PG, Coso OA, Gutkind JS (1996). Suppression of ceramide-mediated programmed cell death by sphingosine-1-phosphate. Nature.

[16] Liu JW, Beckman BS, Foroozesh M (2013). A review of ceramide analogs as potential anticancer agents. Future Med Chem.

[17] Saddoughi SA, Ogretmen B (2013). Diverse Functions of Ceramide in Cancer Cell Death and Proliferation. Adv Cancer Res.

[18] French KJ, Schrecengost RS, Lee BD, Zhuang Y, Smith SN, Eberly JL (2003). Discovery and evaluation of inhibitors of human sphingosine kinase. Cancer Res.

[19] French KJ, Zhuang Y, Maines LW, Gao P, Wang WX, Beljanski V (2010). Pharmacology and Antitumor Activity of ABC294640, a Selective Inhibitor of Sphingosine Kinase-2. J Pharmacol Exp Ther.

[20] Qin ZQ, Dai L, Trillo-Tinoco J, Senkal C, Wang WX, Reske T (2014). Targeting Sphingosine Kinase Induces Apoptosis and Tumor Regression for KSHV-Associated Primary Effusion Lymphoma. Mol Cancer Ther.

[21] Venant H, Rahmaniyan M, Jones EE, Lu P, Lilly MB, Garrett-Mayer E (2015). The Sphingosine Kinase 2 Inhibitor ABC294640 Reduces the Growth of Prostate Cancer Cells and Results in Accumulation of Dihydroceramides In Vitro and In Vivo. Mol Cancer Ther.

[22] Xun C, Chen MB, Qi L, Zhang TN, Peng X, Ning L (2015). Targeting sphingosine kinase 2 (SphK2) by ABC294640 inhibits colorectal cancer cell growth in vitro and in vivo. J Exp Clin Canc Res.

[23] Lewis CS, Voelkel-Johnson C, Smith CD (2016). Suppression of c-Myc and RRM2 expression in pancreatic cancer cells by the sphingosine kinase-2 inhibitor ABC294640. Oncotarget.

[24] Ding XW, Chaiteerakij R, Moser CD, Shaleh H, Boakye J, Chen G (2016). Antitumor effect of the novel sphingosine kinase 2 inhibitor ABC294640 is enhanced by inhibition of autophagy and by sorafenib in human cholangiocarcinoma cells. Oncotarget.

[25] Britten CD, Garrett-Mayer E, Chin SH, Shirai K, Ogretmen B, Bentz TA (2017). A Phase I Study of ABC294640, a First-in-Class Sphingosine Kinase-2 Inhibitor, in Patients with Advanced Solid Tumors. Clin Cancer Res.

[26] Dai L, Trillo-Tinoco J, Bai AP, Chen YH, Bielawski J, Del Valle L (2015). Ceramides promote apoptosis for virus-infected lymphoma cells through induction of ceramide synthases and viral lytic gene expression. Oncotarget.

[27] Cao YY, Qiao J, Lin Z, Zabaleta J, Dai L, Qin ZQ (2017). Up-regulation of tumor suppressor genes by exogenous dhC16-Cer contributes to its anti-cancer activity in primary effusion lymphoma. Oncotarget.

[28] Dai L, Bai AP, Smith CD, Rodriguez PC, Yu FY, Qin ZQ (2017). ABC294640, A Novel Sphingosine Kinase 2 Inhibitor, Induces Oncogenic Virus-Infected Cell Autophagic Death and Represses Tumor Growth. Mol Cancer Ther.

[29] Dai L, Smith CD, Foroozesh M, Miele L, Qin ZQ (2018). The sphingosine kinase 2 inhibitor ABC294640 displays anti-non-small cell lung cancer activities in vitro and in vivo. Int J Cancer.

[30] Dai L, Trillo-Tinoco J, Chen YH, Bonstaff K, Del Valle L, Parsons C (2016). CD147 and downstream ADAMTSs promote the tumorigenicity of Kaposi's sarcoma-associated herpesvirus infected endothelial cells. Oncotarget.

[31] Bielawski J, Szulc ZM, Hannun YA, Bielawska A (2006). Simultaneous quantitative analysis of bioactive sphingolipids by high-performance liquid chromatography-tandem mass spectrometry. Methods.

[32] Kheir F, Zhao MM, Strong MJ, Yu Y, Nanbo A, Flemington EK (2019). Detection of Epstein-Barr Virus Infection in Non-Small Cell Lung Cancer. Cancers.

[33] Mueller S, Hashizume R, Yang XD, Kolkowitz I, Olow AK, Phillips J (2014). Targeting Wee1 for the treatment of pediatric high-grade gliomas. Neuro-Oncology.

[34] Lewis PW, Allis CD (2013). Poisoning the "histone code" in pediatric gliomagenesis. Cell Cycle.

[35] Wan YCE, Liu J, Chan KM (2018). Histone H3 Mutations in Cancer. Curr Pharmacol Rep.

[36] Dan HC, Sun M, Kaneko S, Feldman RI, Nicosia SV, Wang HG (2004). Akt phosphorylation and stabilization of X-linked inhibitor of apoptosis protein (XIAP). J Biol Chem.

[37] Lewin AR, Reid LE, Mcmahon M, Stark GR, Kerr IM (1991). Molecular Analysis of a Human Interferon-Inducible Gene Family. Eur J Biochem.

[38] Deblandre GA, Marinx OP, Evans SS, Majjaj S, Leo O, Caput D (1995). Expression Cloning of an Interferon-Inducible 17-Kda Membrane-Protein Implicated in the Control of Cell-Growth. J Biol Chem.

[39] Yang Y, Lee JH, Kim KY, Song HK, Kim JK, Yoon SR (2005). The interferon-inducible 9-27 gene modulates the susceptibility to natural killer cells and the invasiveness of gastric cancer cells. Cancer Lett.

[40] Brass AL, Huang IC, Benita Y, John SP, Krishnan MN, Feeley EM (2009). The IFITM Proteins Mediate Cellular Resistance to Influenza A H1N1 Virus, West Nile Virus, and Dengue Virus. Cell.

[41] Schwanzel-Fukuda M, Bick D, Pfaff DW (1989). Luteinizing hormone-releasing hormone (LHRH)-expressing cells do not migrate normally in an inherited hypogonadal (Kallmann) syndrome. Molecular Brain Research.

[42] Hannun YA, Obeid LM (2008). Principles of bioactive lipid signalling: lessons from sphingolipids. Nat Rev Mol Cell Bio.

[43] Stiban J, Tidhar R, Futerman AH (2010). Ceramide Synthases: Roles in Cell Physiology and Signaling. Adv Exp Med Biol.

[44] Hannun YA, Obeid LM (2011). Many Ceramides. J Biol Chem.

[45] Wang Z, Wen LJ, Zhu F, Wang YP, Xie Q, Chen ZJ (2017). Overexpression of ceramide synthase 1 increases C18-ceramide and leads to lethal autophagy in human glioma. Oncotarget.

[46] Liu HY, Chen L, Liu JL, Meng HX, Zhang R, Ma L (2017). Co-delivery of tumor-derived exosomes with alpha-galactosylceramide on dendritic cell-based immunotherapy for glioblastoma. Cancer Lett.

[47] Sordillo LA, Sordillo PP, Helson L (2016). Sphingosine Kinase Inhibitors as Maintenance Therapy of Glioblastoma After Ceramide-Induced Response. Anticancer Res.

[48] Balbous A, Cortes U, Guilloteau K, Villalva C, Flamant S, Gaillard A (2014). A mesenchymal glioma stem cell profile is related to clinical outcome. Oncogenesis.

[49] Lee J, Goh SH, Song N, Hwang JA, Nam S, Choi IJ (2012). Overexpression of IFITM1 Has Clinicopathologic Effects on Gastric Cancer and Is Regulated by an Epigenetic Mechanism. Am J Pathol.

[50] Andreu P, Colnot S, Godard C, Laurent-Puig P, Lamarque D, Kahn A (2006). Identification of the IFITM family as a new molecular marker in human colorectal tumors. Cancer Res.

[51] Choy CT, Kim H, Lee JY, Williams DM, Palethorpe D, Fellows G (2014). Anosmin-1 contributes to brain tumor malignancy through integrin signal pathways. Endocr-Relat Cancer.

