# Supplementary material for: Targeting Sphingosine Kinase by ABC294640 against Diffuse Intrinsic Pontine Glioma (DIPG)
Source: J Cancer. 2020 May 22;11(16):4683–91. doi: 10.7150/jca.46269 (PMC7330698; doi:10.7150/jca.46269)
Supplement: Supplementary file 1 — Supplementary figures and tables. [file jcav11p4683s1.pdf]

Supplemental Figure Legends

Figure S1. ABC294640 treatment reduces the proliferation of SF7761 cell line. DIPG cell line SF7761 were treated with the indicated concentrations of ABC294640 or vehicle for 72 h, then photoed by using a contrast microscope.

Figure S2. ABC294640 treatment shows little effects on normal brain cell growth. The cortical neuronal cell-line, HCN-2, were treated with the indicated concentrations of ABC294640 or vehicle for 72 h, then the cell proliferation status was examined using the WST-1 cell proliferation assays (Roche). Error bars represent S.D. for 3 independent experiments.

Figure S3. The distinct morphology between two of DIPG cell lines, SF8628 and SF7761.

Figure S4. The compositions and proportions of ceramide species in ABC294640-treated DIPG cells. Relative compositions and proportions of specific ceramide species were present and compared from vehicle- or ABC294640-treated DIPG cells. Each color representing a specific ceramide species is labeled beside the pie chart.

Figure S1

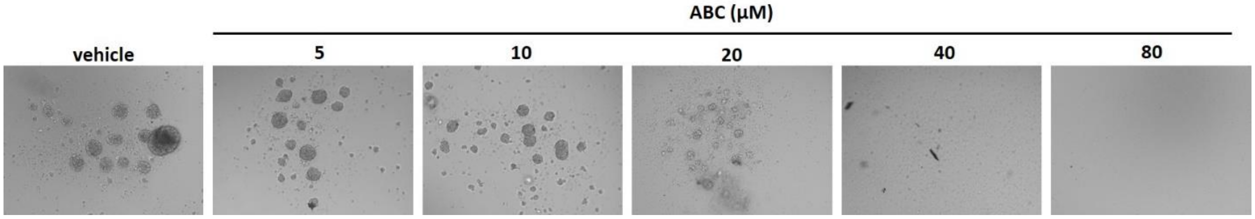

Figure S2

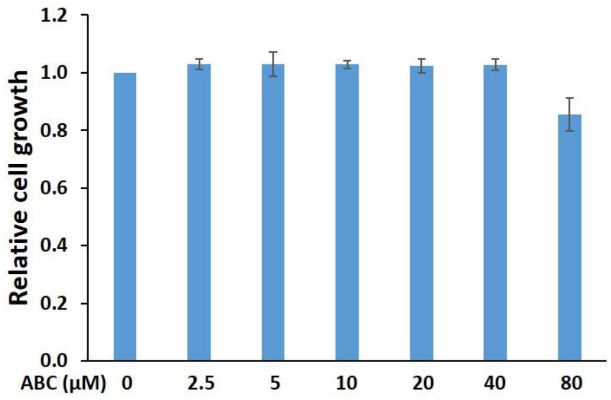

Figure S3

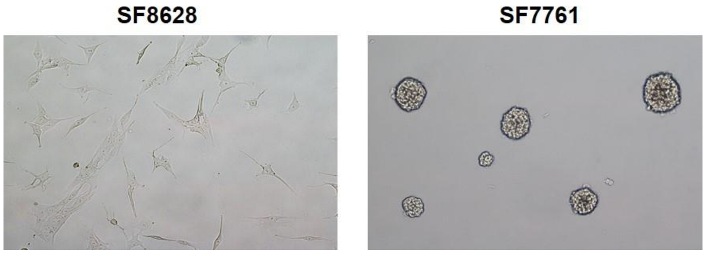

Figure S4

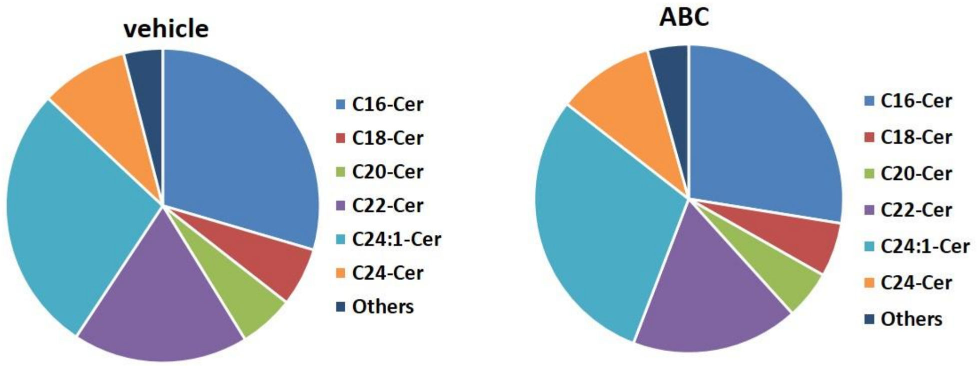
vehicle
C16-Cer
C18-Cer
C20-Cer
C22-Cer
C24:1-Cer
C24-Cer
Others
ABC
C16-Cer
C18-Cer
C20-Cer
C22-Cer
C24:1-Cer
C24-Cer
Others
